# Kennel Disinfectants for *Microsporum canis* and *Trichophyton* sp.

**DOI:** 10.1155/2015/853937

**Published:** 2015-02-11

**Authors:** Karen A. Moriello

**Affiliations:** Department of Medical Sciences, School of Veterinary Medicine, University of Wisconsin-Madison, 2015 Linden Drive West, Madison, WI 53706, USA

## Abstract

The antifungal efficacy of commonly used kennel disinfectants for large surfaces was tested using naturally infective material from untreated animals (*M. canis* and *Trichophyton* sp.) soaked and macerated but unfiltered leaving visible fluorescing hairs and/or scales in the test inoculum to create a robust challenge. Disinfectants included sodium hypochlorite (1 : 32 and 1 : 100), enilconazole (1 : 100), accelerated hydrogen peroxide (1 : 16), potassium peroxymonosulfate (1% and 2%), and calcium hypochlorite “dry bleach.” Disinfectants were tested at a 1 : 10, 1 : 5, and 1 : 1 dilution of test inoculum to disinfectant with a 10 min contact time. Good efficacy was defined as a disinfectant resulting in no growth. Control plates grew >300 colonies of each pathogen per plate. Enilconazole, sodium hypochlorite (all dilutions), accelerated hydrogen peroxide, and 2% potassium peroxymonosulfate (but not 1%) inhibited all growth of both pathogens at 1 : 10, 1 : 5, and 1 : 1 dilutions. Calcium hypochlorite showed no antifungal efficacy (>300 colonies per plate). Enilconazole (1 : 100), sodium hypochlorite (1 : 32 or 1 : 100), accelerated hydrogen peroxide (1 : 16), and 2% potassium peroxymonosulfate are recommended for decontamination of kennels exposed to dermatophyte pathogens.

## 1. Introduction

Environmental disinfection is an important component of the prevention and control of dermatophytosis and is of particular importance in facilities housing large numbers of animals (e.g., animal shelters, boarding kennels, etc.). Many factors need to be considered when selecting a kennel disinfectant including, but not limited to, efficacy, lack of toxicity or irritancy to animals or workers, cost, ease of application, and lack of corrosiveness to surfaces, for example, cages.

Although sodium hypochlorite is a commonly used disinfectant in multianimal facilities, there is increasing interest in using compounds that are inexpensive, easier to use, and less corrosive to cages. One of these compounds is calcium hypochlorite, also referred to as “dry bleach” because it is supplied in pellets and packages. This compound is most commonly used to disinfect foods, swimming pools, and water supplies [1–3]. It is also used in over-the-counter bathroom cleaners and disinfectants and to kill moss and algae. It is an increasingly popular kennel disinfectant because it is automatically diluted and dispensed using a water house, is inexpensive, and is not very corrosive [4].

Although the commercial products do not claim antifungal efficacy, the lay literature on kennel disinfectants often claims it is effective in the control and treatment of dermatophytosis; however published studies to support this could not be found.

One laboratory method used to test the field efficacy of antifungal disinfectants is the isolated infected spore model [5]. In this methodology, infected hairs harvested from untreated animals are used to produce a test inoculum that contains naturally infective material. In this model, the test inoculum is filtered to remove organic debris. A valid criticism of the isolated infective spore test inoculum is that it may not be a robust enough test because it lacks organic material, specifically particulate infective hairs or scales. If the filtering step is omitted the test inoculum contains a marked amount of organic material, that is, small pieces of infective hairs, scales, and debris similar to what would be found on a kennel surface deemed “visibly clean” prior to application of a disinfectant. The objective of this study was to determine the antifungal efficacy of six kennel disinfectants against a robust challenge of naturally infective material.

## 2. Materials and Methods

### 2.1. Test Pathogens

*Microsporum canis* was obtained from kittens with untreated spontaneous infections. For testing of a* Trichophyton *sp. pathogen, infective crusts were obtained from juvenile hedgehogs with spontaneous untreated infections (i.e.,* T. erinacei*).

### 2.2. Preparation of Infective Spore Suspensions

Infective spore suspensions were prepared using a modification of previously published method [5]. Briefly, naturally infective material was soaked in sterile water for 15 min and minced with a sterile scalpel blade three times. Using a tissue macerator the suspension was macerated until an opaque solution was obtained. For this study, the solution was not filtered and hair and scales were grossly visible. Wood's lamp examination of the* M. canis* spore suspension revealed large numbers of fluorescing particles and microscopic examination of the* Trichophyton* sp. suspension revealed intact crusts and identifiable scales.

### 2.3. Fungal Cultures

Mycosel Agar (Becton Dickinson, Cockeyville, MD, USA) was used for fungal cultures. Plates were incubated at 30°C and examined daily for growth for 14 days. Colony forming units (cfu) for each plate were determined as the maximum number of colonies at day 14 of culture. Potential pathogens were identified microscopically using established morphological criteria.

### 2.4. Kennel Disinfectants

Disinfectants tested included 5.5% sodium hypochlorite diluted 1 : 32 and 1 : 100, accelerated hydrogen peroxide at 1 : 16 (Accel Concentrate 4.25%, Virox Technologies, Oakville, Ontario, Canada), potassium peroxymonosulfate at 1% and 2% (Trifectant: Vetoquinol, Fort Worth, TX), and enilconazole at 1 : 100 (Clinafarm, Schering Plough Animal Health, Union, New Jersey) and calcium hypochlorite (Wysiwash, Wysiwash, South Daytona, FL, USA). For calcium hypochlorite one liter of test solution was collected midstream after allowing the hose to run for 3 min. All test solutions were prepared fresh and used within 3 h. Sterile distilled water and 5.5% sodium hypochlorite (1 : 10 dilution) were used as untreated and treated controls, respectively.

### 2.5. Testing Protocol

Infective spore suspensions were tested at a 1 : 10, 1 : 5, and 1 : 1 dilution of spores to disinfectant with a contact time of 10 min. Testing solutions were vortexed four times (0 min, 5 min, 9 min, and 10 min) during the contact time to ensure adequate exposure of spores to disinfectant. Four 100 *μ*L aliquots of each test suspension were inoculated on fungal culture plates by spreading the suspension evenly on the surface with a sterile loop. Stock suspensions were repeatedly vortexed before and between samplings to ensure fungal spores and hairs did not settle in the test tube. All testing was done in triplicate. Disinfectants lacking antifungal efficacy were retested using isolated infected spores, that is, filtered test inoculum.

### 2.6. Data Analysis

Descriptive data was collected. The number of colony forming units per plate was counted and presented as the mean ± standard deviation. For the purposes of this study, good efficacy was defined as a disinfectant that produced no growth.

## 3. Results and Discussion

A 100 *μ*L inoculum of each pathogen grew too many to count colonies per plate. Serial dilutions estimated the 5 × 10^5^ and 8 × 10^6^ infective spores per mL for* M. canis* and* Trichophyton* sp., respectively. However this number likely underestimated the test inoculum since it cannot account for the spore challenge in particulate hair or scales. Pre-, mid-, and postexperiment 100 *μ*L inoculums grew >300 cfu/plate for both pathogens. Enilconazole, AHP, 2% potassium peroxymonosulfate, and all dilutions of sodium hypochlorite were 100% fungicidal (Table 1) against both pathogens. Calcium hypochlorite lacked fungicidal activity (Table 1) in this test model and when retested using isolated infected spores (i.e., filtered test inoculum). 1% potassium peroxymonosulfate was not considered to have good efficacy compared to 2% potassium peroxymonosulfate.

In this study, calcium hypochlorite demonstrated no fungicidal activity against naturally infective material. This is most likely due to the fact that this product is primarily an algaecide.

Decontamination of surfaces exposed to dermatophytes requires a “hard clean.” Specifically, all gross material should be mechanically removed and the area washed with a detergent until visibly clean, rinsed to remove residual detergents and excess water removed to prevent dilution of the disinfectant, and then finally sprayed with a disinfectant. Disinfectants are intended to kill any remaining spores not removed via mechanical cleaning. With respect to this, it is important to note two things. First, there was a strict 10 min contact time. Second, although the test suspensions contained visible hair and skin debris no other organic material was present (e.g., feces, serum, and food) that could prevent contact between the infective material and the disinfectant. Although these disinfectants were highly efficacious against a robust spore challenge, proper surface preparation is still a necessity for good sanitation.

One of the reasons this study was conducted was to answer anecdotal comments that original isolated infective spore model results were not representative of the “real world” because hair and debris were removed. The findings in this study are similar to those previously published using filtered isolated infective spores in either suspension tests or textile disinfectant testing [5–7]. This is supporting evidence that the original isolated infective spore model is a robust challenge and that data using that model is valid. The advantage of the isolated infective spore model over the procedure used in this study was that less naturally infective material is needed.

## 4. Conclusions and Clinical Relevance

In practice, these findings suggest that if obvious organic debris is removed and the surface is visibly clean, the use of sodium hypochlorite (1 : 32 and 1 : 100), enilconazole 1 : 16, 2% potassium peroxymonosulfate, or accelerated hydrogen peroxide 1 : 16 with a 10 min contact time is an efficacious disinfectant for any residual remaining infective material of dermatophytosis.

## Figures and Tables

**Table 1:** Mean number of colony forming units after exposure to disinfectants.

	*M. canis *			*Trichophyton* sp.		
	1 : 10	1 : 5	1 : 1	1 : 10	1 : 5	1 : 1
Water	>100	>100	>100	>100	>100	>100
Sodium hypochlorite 1 : 10	0	0	0	0	0	0
Sodium hypochlorite 1 : 32	0	0	0	0	0	0
Sodium hypochlorite 1 : 100	0	0	0	0	0	0
AHP 1 : 16	0	0	0	0	0	0
Enilconazole 1 : 100	0	0	0	0	0	0
2% potassium peroxymonosulfate	0	0	0	0	0	0
1% potassium peroxymonosulfate	4 ± 5	15 ± 6	23 ± 3	0	2 ± 3	7 ± 4
Calcium hypochlorite	>100	>100	>100	>100	>100	>100

Mean of the agar plates ± standard deviation.

AHP: accelerated hydrogen peroxide (Accel).
